# Asparagine Is a Critical Limiting Metabolite for Vaccinia Virus Protein Synthesis during Glutamine Deprivation

**DOI:** 10.1128/JVI.01834-18

**Published:** 2019-06-14

**Authors:** Anil Pant, Shuai Cao, Zhilong Yang

**Affiliations:** a Division of Biology, Kansas State University, Manhattan, Kansas, USA; University of Illinois at Urbana Champaign

**Keywords:** asparagine, glutamine, metabolic profiling, metabolism, poxvirus, protein synthesis, vaccinia virus

## Abstract

Viruses actively interact with host metabolism because viral replication relies on host cells to provide nutrients and energy. Vaccinia virus (VACV; the prototype poxvirus) prefers glutamine to glucose for efficient replication to the extent that VACV replication is hindered in glutamine-free medium. Remarkably, our data show that VACV replication can be fully rescued from glutamine depletion by asparagine supplementation. By global metabolic profiling, as well as genetic and chemical manipulation of the asparagine supply, we provide evidence demonstrating that the production of asparagine, which exclusively requires glutamine for biosynthesis, accounts for VACV’s preference of glutamine to glucose rather than glutamine’s superiority over glucose in feeding the tricarboxylic acid (TCA) cycle. Furthermore, we show that sufficient asparagine supply is required for efficient VACV protein synthesis. Our study highlights that the asparagine supply, the regulation of which has been evolutionarily tailored in mammalian cells, presents a critical barrier to VACV replication due to a high asparagine content of viral proteins and a rapid demand of viral protein synthesis. The identification of asparagine availability as a critical limiting factor for efficient VACV replication suggests a new direction of antiviral strategy development.

**IMPORTANCE** Viruses rely on their infected host cells to provide nutrients and energy for replication. Vaccinia virus, the prototypic member of the poxviruses, which comprise many significant human and animal pathogens, prefers glutamine to glucose for efficient replication. Here, we show that the preference is not because glutamine is superior to glucose as the carbon source to fuel the tricarboxylic acid cycle for vaccinia virus replication. Rather interestingly, the preference is because the asparagine supply for efficient viral protein synthesis becomes limited in the absence of glutamine, which is necessary for asparagine biosynthesis. We provide further genetic and chemical evidence to demonstrate that asparagine availability plays a critical role in efficient vaccinia virus replication. This discovery identifies a weakness of vaccinia virus and suggests a possible direction to intervene in poxvirus infection.

## Abstract (precis)

Viruses rely on their infected host cells to provide nutrients and energy for replication. Vaccinia virus, the prototypic member of the poxviruses, which comprise many significant human and animal pathogens, prefers glutamine to glucose for efficient replication. Here, we show that the preference is not because glutamine is superior to glucose as the carbon source to fuel the tricarboxylic acid cycle for vaccinia virus replication. Rather interestingly, the preference is because the asparagine supply for efficient viral protein synthesis becomes limited in the absence of glutamine, which is necessary for asparagine biosynthesis. We provide further genetic and chemical evidence to demonstrate that asparagine availability plays a critical role in efficient vaccinia virus replication. This discovery identifies a weakness of vaccinia virus and suggests a possible direction to intervene in poxvirus infection.

## INTRODUCTION

Viruses do not have metabolic machinery, so viral replication relies on the host for a supply of nutrients and energy. Unsurprisingly, metabolism is a crucial interface of virus-host interactions. Many viral infections are characterized as being heavily dependent on particular metabolites (e.g., glutamine, glucose, or fatty acids) for optimal replication. Many viruses also induce alterations in metabolic pathways, such as those for glycolysis, synthesis of fatty acids and nucleotides, and energy metabolism. The abilities of viruses to make these alterations often shape the outcome of viral infections (1–4).

Vaccinia virus (VACV) is the prototype poxvirus, with a large double-stranded DNA genome that encodes more than 200 annotated genes (5, 6). Many poxviruses cause fatal diseases, such as variola virus-induced smallpox, which is one of the most devastating infectious diseases in human history. Although eradicated in nature, smallpox is still a valid national security concern due to potential unregistered stocks or *de novo* synthesis of live variola virus (7–9). Moreover, other poxviruses cause human and animal diseases. On the other hand, poxviruses are practically useful as oncolytic agents for cancer treatments and as vectors for vaccine development and recombinant protein production (10–13). For efficient VACV replication in cell culture, VACV prefers glutamine to glucose; the depletion of glutamine, but not glucose, from culture medium significantly decreases VACV production (14, 15). In line with this finding, VACV infection upregulates glutamine metabolism (16). Nevertheless, why VACV prefers glutamine to glucose for replication remains elusive.

**FIG 1:**
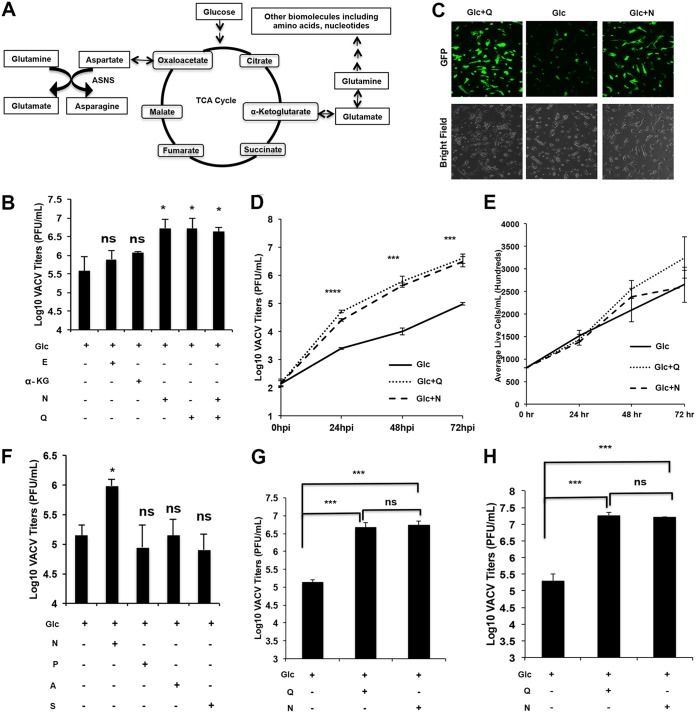
Asparagine fully rescues VACV replication from glutamine depletion. (A) Schematic of the role of glutamine in the TCA cycle and biomolecule synthesis. Note that asparagine exclusively requires glutamine for its biosynthesis. (B) Asparagine fully rescues VACV replication from glutamine depletion, while α-KG and glutamate do not. HFFs were infected with VACV at an MOI of 2 in medium containing 1 g/liter glucose (Glc), 2 mM glutamine (Q), 2 mM asparagine (N), 7 mM α-KG, or 5 mM glutamate (E), as indicated. VACV titers were measured by a plaque assay at 24 hpi. (C) Asparagine rescues green fluorescent protein (GFP) expression from recombinant VACV in the absence of glutamine. HFFs were infected with a recombinant VACV encoding a GFP gene at an MOI of 2 in the indicated medium. GFP expression was observed under a microscope at 24 hpi. (D) Asparagine rescues VACV growth kinetics from glutamine depletion. HFFs were infected with VACV at an MOI of 0.001 in medium containing the indicated nutrients. VACV titers were measured by a plaque assay at the indicated times postinfection. (E) HFF proliferation is not affected in different growth media. Equal numbers of HFFs were seeded into the indicated media. The cell numbers were counted over a 72-h period of using a hemocytometer. (F) Proline (P), alanine (A), and serine (S) cannot rescue VACV replication from glutamine depletion. Experiments were carried out similarly to those shown in panel B, with 5 mM proline, 1 mM alanine, or 1 mM serine used. (G) Asparagine rescues VACV replication from glutamine depletion in BS-C-1 cells. BS-C-1 cells were infected with VACV at an MOI of 2 in the indicated media, and virus titers were measured at 24 hpi. (H) BS-C-1 cells were infected with VACV at an MOI of 0.01 in the indicated media, and the virus titers were measured at 48 hpi by a plaque assay. Error bars represent the standard deviation of at least three biological replicates. ns, *P* > 0.05; *, *P* ≤ 0.05; ***, *P* ≤ 0.001; ****, *P* ≤ 0.0001.

Glutamine is a nonessential amino acid that is abundantly utilized by mammalian cells beyond its role as a protein building block (17). Glutamine feeds the tricarboxylic acid (TCA) cycle (Fig. 1A) through glutamate and alpha-ketoglutarate (α-KG) in a process known as anaplerosis (18–20). Glutamine also acts as a biosynthetic precursor for many molecules, including amino acids, nucleotides, and fatty acids (21, 22). Although several nonessential amino acids require intermediates of glutamine metabolism for *de novo* biosynthesis, only asparagine biosynthesis exclusively depends on glutamine because the amination of the synthesis reaction requires glutamine (23, 24). The biosynthesis of asparagine using glutamine is catalyzed by the enzyme asparagine synthetase (ASNS) (25, 26).

A new and growing body of work suggests that asparagine is more than just a polypeptide subunit. It is essential in coordinating overall protein synthesis, cellular responses to amino acid homeostasis, and metabolic availability during biological processes and disease development. For example, asparagine acts as a metabolic regulator of TCA cycle intermediates and the cellular supply of nitrogen (which supports the synthesis of nonessential amino acids); for cancer cells, asparagine bioavailability is essential for survival, proliferation, and tumor development (23, 24, 27, 28). Asparagine is also important for supporting Kaposi’s sarcoma-associated herpesvirus (KSHV) transformed cancer cell proliferation due to its critical role in nucleotide biosynthesis during glutamine depletion (29). However, the role of asparagine availability in virus replication has not been explored.

In the current study, we show that asparagine is a limiting metabolite for VACV replication through its critical role in VACV protein synthesis. In contrast to the generic paradigm that glutamine is superior to glucose in fueling the TCA cycle, we show that the preference for glutamine reflects the requirement of sufficient asparagine supply during replication. Indeed, interfering with asparagine metabolism severely impairs VACV replication, highlighting the importance of asparagine availability during the VACV life cycle. Our findings demonstrate an essential role of asparagine availability for efficient VACV replication. Understanding this critical host-dependent barrier to VACV replication might not only spur the development of new, host-oriented antiviral therapies but also improve the development of poxviruses as therapeutic tools.

(This article was submitted to an online preprint archive [30].)

## RESULTS

### Asparagine fully rescues VACV replication from glutamine depletion.

To test why VACV prefers glutamine to glucose for efficient replication, we examined whether α-KG and glutamate—the products of glutaminolysis that feed the TCA cycle (Fig. 1A)—could rescue VACV replication from glutamine depletion. Measuring virus titers showed that α-KG and glutamate supplementation only partially rescued VACV replication in the absence of glutamine (Fig. 1B), in agreement with results of earlier studies (14, 15). This indicates that while anaplerosis of the TCA cycle is important, this function of glutamine is not responsible for its superiority to glucose in promoting VACV replication.

Notably, VACV replication was fully rescued from glutamine depletion when asparagine was added to the medium (Fig. 1B). In contrast, when the medium contained glutamine, adding asparagine did not boost viral titers, suggesting that growth could be equally rescued by either glutamine or asparagine. Moreover, asparagine rescued green fluorescent protein (GFP) expression from a recombinant VACV expressing GFP in the absence of glutamine (Fig. 1C).

VACV replication kinetics over a 72-h period using an initial VACV multiplicity of infection (MOI) of 0.001 in the glutamine-free medium were also consistently rescued by asparagine (Fig. 1D). The 72-h proliferation rate of human foreskin fibroblasts (HFFs) differed little from that of cells grown in medium containing glucose only or glucose plus asparagine (Fig. 1E), suggesting that the difference in VACV titers is not due to altered HFF proliferation. Other nonessential amino acids that can be synthesized from glutamine but were not present in the cell culture medium (e.g., proline, alanine, and serine), did not rescue VACV replication from glutamine deficiency (Fig. 1F). We also tested the effect of asparagine in supporting VACV replication upon glutamine depletion in BS-C-1 (a monkey kidney epithelial cell line) cells. Similar to the results in HFFs, asparagine fully rescued VACV titers from glutamine depletion in BS-C-1 cells (Fig. 1G and H). Together, these results demonstrate that asparagine can rescue VACV replication from glutamine depletion.

### Asparagine does not enhance TCA cycle activities during VACV infection.

**FIG 2:**
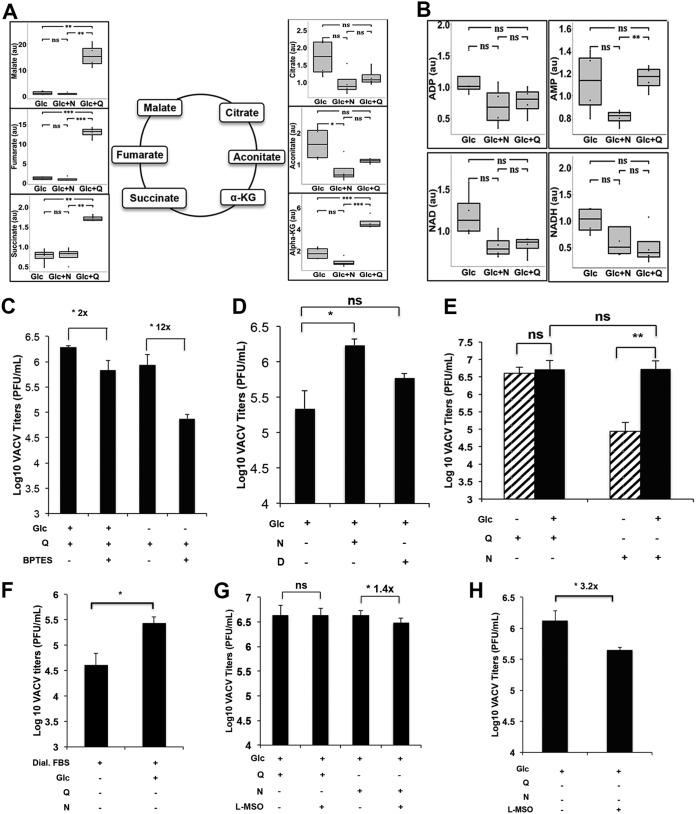
Asparagine supplementation does not enhance TCA cycle activities under glutamine-depleted conditions during VACV infection. (A) Asparagine addition does not recapitulate glutamine’s effect on TCA cycle activities. Levels of TCA cycle intermediates in HFFs infected with VACV (MOI of 3) for 8 h, in medium containing glucose (Glc), glucose plus asparagine (Glc+N), or glucose plus glutamine (Glc+Q), were determined by global metabolic profiling. (B) Levels of oxidative phosphorylation intermediates in VACV-infected HFFs are not significantly different (from the same metabolic profiling as described in the legend to panel A). (C) Inhibiting glutaminase activity more severely affected VACV replication in the absence of glucose. HFFs were infected with VACV, at an MOI of 2 for 24 h, in medium containing 10 μM bis-2-(5-phenylacetamido-1,3,4-thiadiazol-2-yl)ethyl sulfide (BPTES) or dimethyl sulfoxide (DMSO) (control). The numbers indicate the fold change in VACV titer compared to that with DMSO treatment. (D) Aspartate is not as efficient as asparagine in supporting VACV replication. VACV titers in HFFs infected with VACV, at an MOI of 2 for 24 h, in medium with glucose (Glc), asparagine (N), or aspartate (D) were measured by a plaque assay. (E) Rescue of VACV replication from glutamine depletion requires the presence of glucose. HFFs were infected with VACV, at an MOI of 2 for 24 h, in different media in the presence or absence of glucose as indicated. VACV titer was measured by a plaque assay. (F) VACV replication decreases when glucose is not added to the culture medium in the absence of glutamine. HFFs were infected with VACV, at an MOI of 2 for 24 h, in medium with 2% dialyzed fetal bovine serum (FBS) in the presence or absence of glucose (Glc). VACV titer was measured by a plaque assay. (G) Inhibition of glutamine synthetase only slightly affected VACV replication in the presence of glutamine or asparagine. HFFs were infected with VACV at an MOI of 2 for 24 h in indicated medium containing 2 mM l-methionine sulfoximine (L-MSO) or DMSO. VACV titers were determined by plaque assay. (H) L-MSO has a more significant effect in VACV replication in the absence of glutamine or asparagine. Experiments performed as described in the legend to panel G in medium containing glucose only. Error bars represent the standard deviation of at least three replicates. ns, *P* > 0.05; *, *P* ≤ 0.05; **, *P* ≤ 0.01; ***, *P* ≤ 0.001. The numbers above the bars represent fold changes.

Under the glutamine-free condition, asparagine might rescue VACV replication by improving anaplerosis of the TCA cycle. To test this idea, VACV-infected HFFs were profiled for metabolic activities under three different conditions, glucose, glucose plus glutamine, and glucose plus asparagine (Table S1). Glucose plus glutamine significantly enhanced the concentrations of several TCA cycle intermediates (α-KG, succinate, fumarate, and malate) compared to that under the glucose-only condition, while the addition of asparagine did not (Fig. 2A). Even in the absence of glutamine, glucose was sufficient to maintain the levels of oxidative phosphorylation intermediates required for ATP production (Fig. 2B). These results show that rescue of VACV replication by asparagine in the glutamine-deficient medium is not driven by enhancement of the TCA cycle, and that glucose can support enough TCA cycle activities for VACV infection. Additionally, when glutaminase activity is inhibited with bis-2-(5-phenylacetamido-1,3,4-thiadiazol-2-yl)ethyl sulfide (BPTES), VACV titers decreased by only 2-fold in the presence of glucose but decreased by 12-fold in the absence of glucose (glutamine was present in both conditions; Fig. 2C). Together, these results indicate that asparagine-mediated rescue of VACV replication is not due to enhanced TCA cycle activities under glutamine-depleted conditions.

**FIG 3:**
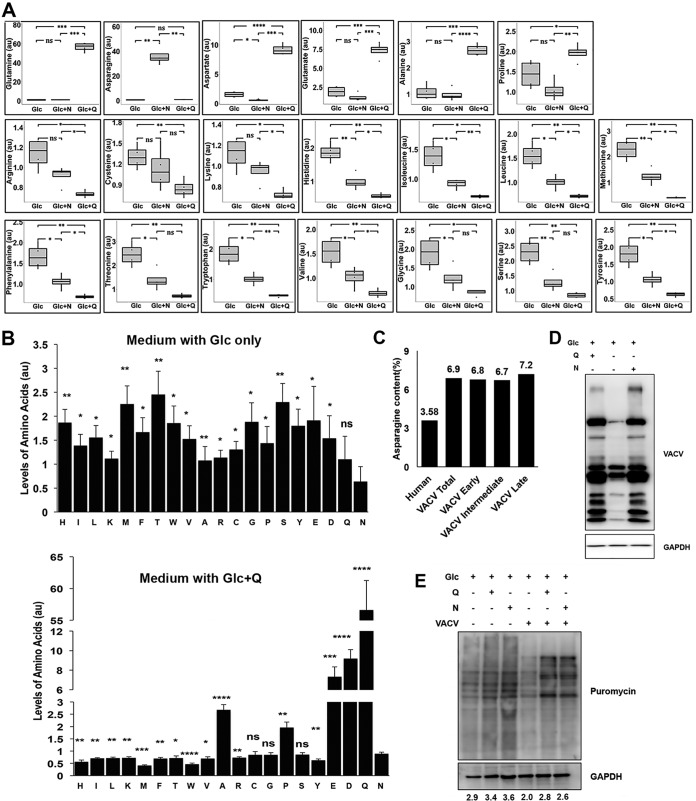
Asparagine rescues VACV protein synthesis from glutamine depletion. (A) Addition of asparagine decreases accumulation of most amino acids under glutamine-depleted conditions. Relative levels of amino acids in HFFs infected with VACV at an MOI of 3 for 8 h in medium, as described in Fig. 2A, were determined by global metabolic profiling. (B) Upon glutamine depletion, asparagine is the least abundant amino acid in VACV-infected cells. Upon glutamine repletion, asparagine is one of the most abundant amino acids. The levels of amino acids in HFFs infected with VACV at an MOI of 3 for 8 h in media with glucose (top) and glucose plus glutamine (bottom) were determined by global metabolic profiling. Statistical significance is shown by comparison to asparagine level. (C) Asparagine contents of VACV and human genome-encoded proteins calculated by ExPASy ProtParam tool. The numbers above the bar indicate the percentage of asparagine content. (D) Asparagine rescues VACV protein synthesis from glutamine depletion. Western blotting was carried out in HFFs infected with VACV at an MOI of 2 for 24 h in the indicated medium. Glyceraldehyde-3-phosphate dehydrogenase (GAPDH) was used as a loading control. (E) Rescue of nascent protein synthesis by asparagine under glutamine-depleted condition. HFFs were infected with VACV at an MOI of 2 or mock infected for 16 h in the indicated medium. Cells were treated with 10 μg/ml puromycin for 10 min before collection followed by Western blotting using antipuromycin and anti-GAPDH antibodies (the latter as a loading control). The numbers indicate GAPDH-normalized puromycin intensities. Error bars represent the standard deviation of four biological replicates. ns, *P* > 0.05; *, *P* ≤ 0.05; **, *P* ≤ 0.01; ***, *P* ≤ 0.001; ****, *P* ≤ 0.0001.

Our interpretation is also supported by the fact that the TCA cycle is not directly fed by asparagine converting to aspartate in VACV-infected cells. Adding aspartate to glutamine-deficient medium rescued only low levels of VACV replication (Fig. 2D), and adding asparagine did not elevate aspartate concentration (Fig. 3A). This is consistent with the fact that in mammalian cells asparaginase does not actively convert asparagine to aspartate (28). Notably, glutamine itself could support VACV replication even in the absence of glucose, while the asparagine-mediated rescue of VACV replication from the glutamine-depleted condition required glucose in the medium (Fig. 2E). Moreover, VACV titer was significantly lower when glucose was not added to the glutamine-depleted medium (Fig. 2F). These results suggest that glutamine can provide functions of both glucose and asparagine during VACV infection.

For VACV-infected HFFs cultured in glutamine-deficient medium, adding asparagine did not increase the glutamine and glutamate concentration either (Fig. 3A). Moreover, using l-methionine sulfoximine (L-MSO) to inhibit *de novo* glutamine synthesis only minimally reduced VACV replication (by 1.4-fold) when the glutamine-deficient medium was supplemented with asparagine (Fig. 2G), showing that VACV replication is not rescued because asparagine supplementation increases glutamine or glutamate to feed the TCA cycle. L-MSO treatment decreased VACV titer by 3.2-fold in glucose-only medium (Fig. 2H), indicating a stronger inhibitory effect of L-MSO on VACV replication when *de novo* glutamine synthesis is suppressed in the absence of exogenous glutamine.

### Asparagine rescues VACV protein synthesis from glutamine depletion.

Notably, our global metabolic profiling data showed that most amino acids (14 out of 20) accumulated in cultures with glucose only, which led to an amino acid imbalance compared to cultures containing glutamine and glucose (Fig. 3A). Within infected cells cultured in glucose-only medium, amino acids whose biosynthesis is closely tied to glutamine concentration (i.e., alanine, proline, aspartate, glutamate, and asparagine) (31, 32) had a lower or similar concentration (Fig. 3A). Among the five amino acids, asparagine is the only amino acid that exclusively requires glutamine for its biosynthesis and is also the only amino acid that fully rescues VACV replication from glutamine depletion. Remarkably, adding asparagine significantly decreased the accumulation of most amino acids under the glutamine-depleted condition (Fig. 3A). Moreover, asparagine concentration was significantly lower than those of other amino acids, except for that of glutamine in glucose-only condition, while its level was significantly higher than or similar to those of most other amino acids when glutamine is present (Fig. 3B).

These results prompted us to hypothesize that asparagine availability is a critical limiting factor in maintaining amino acid balance for efficient protein synthesis in VACV-infected cells. This implies that, without glutamine, the rate of protein synthesis is suppressed by a low asparagine supply that cannot support the acute demand for nascent protein synthesis during the brief time window of VACV replication; exogenous asparagine can correct this amino acid imbalance. Higher demand for asparagine in VACV-infected cells can also be attributed to a 93% higher asparagine content in VACV-encoded proteins than that in human genome-encoded proteins (Fig. 3C). Asparagine content in VACV late proteins, which are expressed at very high levels for viral particles (33, 34), is 101% higher than that in human proteins (Fig. 3C). The hypothesis is supported by the result that VACV protein levels were much lower in cells cultured with glucose only compared to those in cells cultured with glutamine or asparagine (Fig. 3D). There was also less nascent cellular protein synthesis in uninfected HFFs grown in glucose-only medium (Fig. 3E). However, because there are preexisting cellular proteins, glutamine depletion in the absence of asparagine did not affect uninfected HFF proliferation (Fig. 1E). VACV infection directs cellular machinery to synthesize viral proteins, as can be seen in the different patterns of newly synthesized proteins with or without VACV infection (Fig. 3E). The level of nascent viral protein synthesis was lower in VACV-infected HFFs in medium containing glucose only (Fig. 3E). Because all viral proteins need to be newly synthesized after infection, the negative effect on protein synthesis in glucose-only medium presents a severe impact on VACV replication.

**FIG 4:**
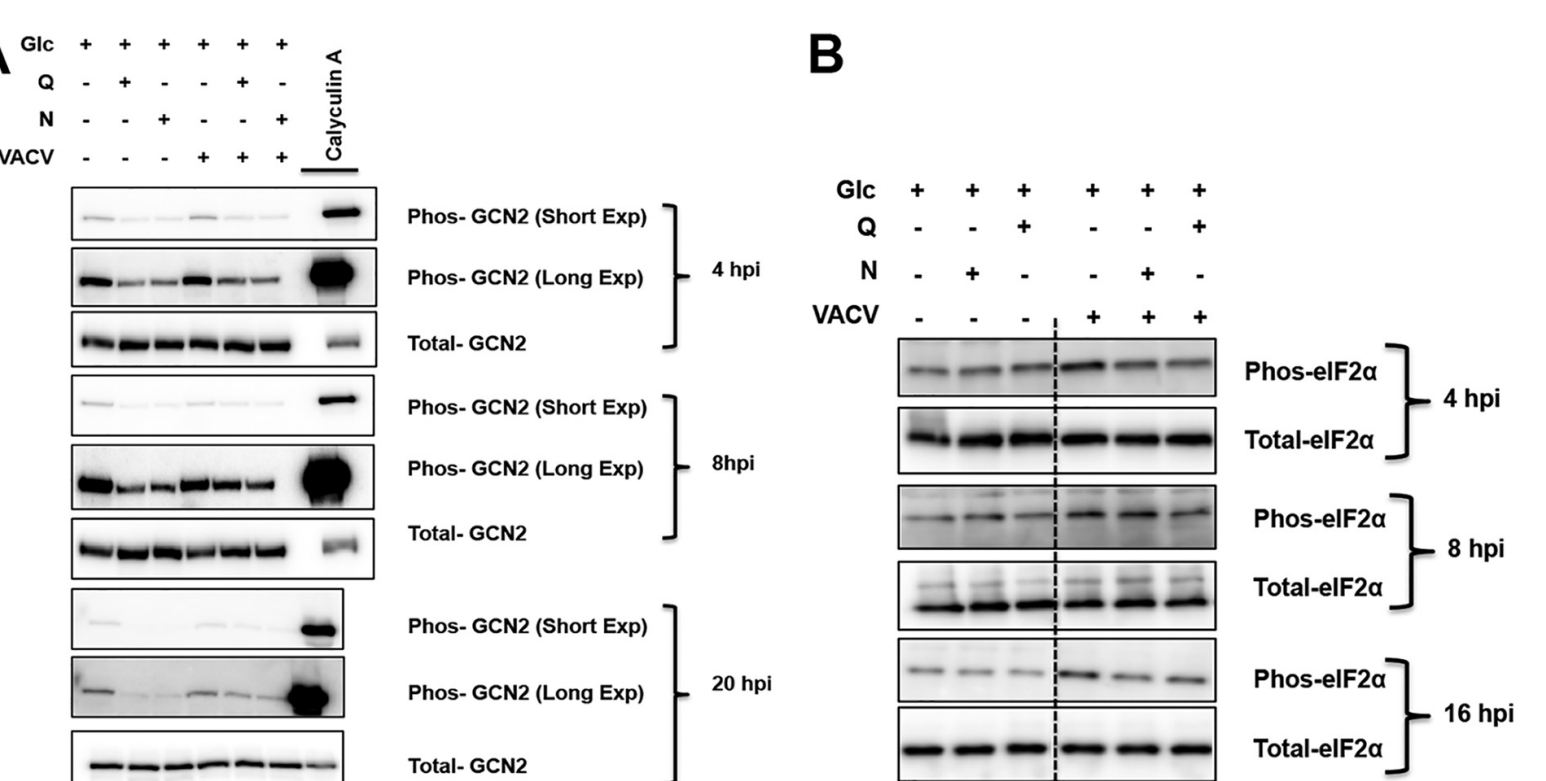
GCN2 and eIF2α phosphorylation are affected by different growth conditions in VACV-infected cells. (A) GCN2 phosphorylation in HFFs infected with VACV or mock infected in different media. HFFs were infected with VACV for 4 h, 8 h, and 20 h in the indicated media. The levels of proteins were determined by Western blotting using indicated antibodies. HFFs were treated with 100 nM calyculin A for 30 min as a positive control for GCN2 phosphorylation. (B) eIF2α phosphorylation in HFFs infected with VACV or mock infected in different media. HFFs were infected with VACV for 4 h, 8 h, and 16 h in the indicated media. The levels of proteins were determined by Western blotting using indicated antibodies. The blots of uninfected and VACV-infected cell lysates were from lanes on the same gel separated by a vertical dashed line.

GCN2 is a metabolic stress-sensing protein kinase that senses amino acid availability and phosphorylates eIF2α to suppress protein synthesis during amino acid deficiency (35, 36). In addition to the effect of a limited supply of asparagine on protein synthesis, there is a possibility that the amino acid imbalance stimulates GCN2/eIF2α phosphorylation to decrease protein synthesis. GCN2 phosphorylation increased in cells grown in glucose-only medium over the course of viral infection and in mock-infected cells, although the phosphorylation levels were significantly lower than those under calyculin A treatment, which served as the positive control of GCN2 phosphorylation (Fig. 4A). Interestingly, eIF2α phosphorylation was not affected in uninfected HFFs in different culture media, while eIF2α phosphorylation increased in cells grown in glucose-only medium during VACV infection (Fig. 4B). Although the changes in eIF2α phosphorylation were not high in different media during VACV replication, their impact should not be neglected, as a small increase in eIF2α phosphorylation may cause significant suppression in mRNA translation (37).

### Asparagine rescues VACV postreplicative mRNA translation from glutamine deficiency.

**FIG 5:**
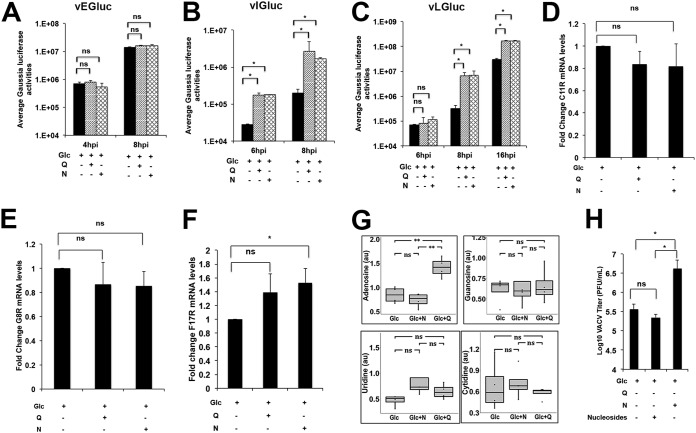
Asparagine rescues VACV postreplicative mRNA translation from glutamine depletion. (A to C) Efficient VACV intermediate and late gene expression, but not early gene expression, requires the presence of asparagine in the glutamine-depleted medium. HFFs infected with VACV that expressed Gaussia luciferase under early (vEGLuc; A), intermediate (vIGLuc; B), and late (vLGLuc; C) promoters, respectively, in indicated medium, followed by Gaussia luciferase activity measurement at indicated times. (D to F) Effects of asparagine or glutamine in glucose-only medium on VACV early (C11R; D), intermediate (G8R; E), and late (F17R; F) mRNA levels. RNA was extracted from HFFs infected with VACV at an MOI of 2, and reverse transcription-quantitative PCR (qRT-PCR) analysis was performed. (G) Asparagine addition does not increase levels of nucleosides in the glutamine-depleted medium. Relative levels of nucleosides in HFFs infected with VACV at an MOI of 3 for 8 h in medium containing glucose (Glc), glucose plus asparagine (Glc+N), or glucose plus glutamine (Glc+Q), were determined by global metabolic profiling. (H) Addition of exogenous nucleosides to the glutamine-depleted medium does not rescue VACV replication. HFFs were infected with VACV at an MOI of 2 in the indicated medium in the presence or absence of 1× nucleosides for 24 h, followed by VACV titer measurement using a plaque assay. Error bars represent the standard deviation of at least three biological replicates. ns, *P* > 0.05; *, *P* ≤ 0.05; **, *P* ≤ 0.01.

VACV genes are expressed in a cascade fashion. Upon entry, VACV early genes are immediately expressed, then DNA is replicated and intermediate genes are expressed, followed by late genes (5). To find out which stage of viral replication was affected, HFFs were infected with one of three reporter VACVs that encode a secreted Gaussia luciferase gene under viral early (vEGluc), intermediate (vIGluc), and late (vLGluc) promoters. Viral gene expression was measured by Gaussia luciferase activities in cell culture medium. Under all three conditions, early gene expression was similar, but VACV intermediate and late gene expressions were significantly higher in medium containing asparagine or glutamine (Fig. 5A to C). The promoters of each class of VACV genes share the same transcription mechanism and use the same transcription factors (5). Therefore, the C11, G8, and F17 mRNA levels can reflect the mRNA levels of VACV early, intermediate, and late mRNAs. reverse transcription-quantitative PCR (qRT-PCR) profiled VACV early (C11R, 4 hours postinfection [hpi]), intermediate (G8R, 6 hpi), and late (F17R, 12 hpi) gene mRNA levels under different nutrient conditions (Fig. 5D to F) (5). Unsurprisingly, the levels of early viral mRNAs were not affected by nutrient conditions (Fig. 5D). VACV intermediate mRNA levels did not differ either, and asparagine or glutamine only mildly increased viral late mRNA levels by less than 1.5-fold (Fig. 5EF). Because VACV intermediate and late mRNA synthesis rely on viral DNA replication and viral DNA replication factors are mostly encoded by viral early genes (6, 38), the glucose-only medium in the absence of glutamine and asparagine is not expected to have a significant effect on VACV DNA replication. These results indicate that asparagine rescues VACV protein synthesis from glutamine deficiency mainly at the postreplicative (both intermediate and late) mRNA translation stage.

There was a significant increase in adenosine levels in the presence of glutamine, likely because glutaminolysis can contribute to nucleotide synthesis (Fig. 5G) (21, 22). However, our metabolic profiling of VACV-infected cells grown with glucose or asparagine supplementation showed that they had similar nucleoside concentrations (Fig. 5G). Accordingly, adding nucleosides to glutamine-depleted medium did not rescue VACV replication (Fig. 5H). These results support the observations that asparagine has no or only small effects on VACV RNA synthesis.

### ASNS knockdown impairs VACV replication.

**FIG 6:**
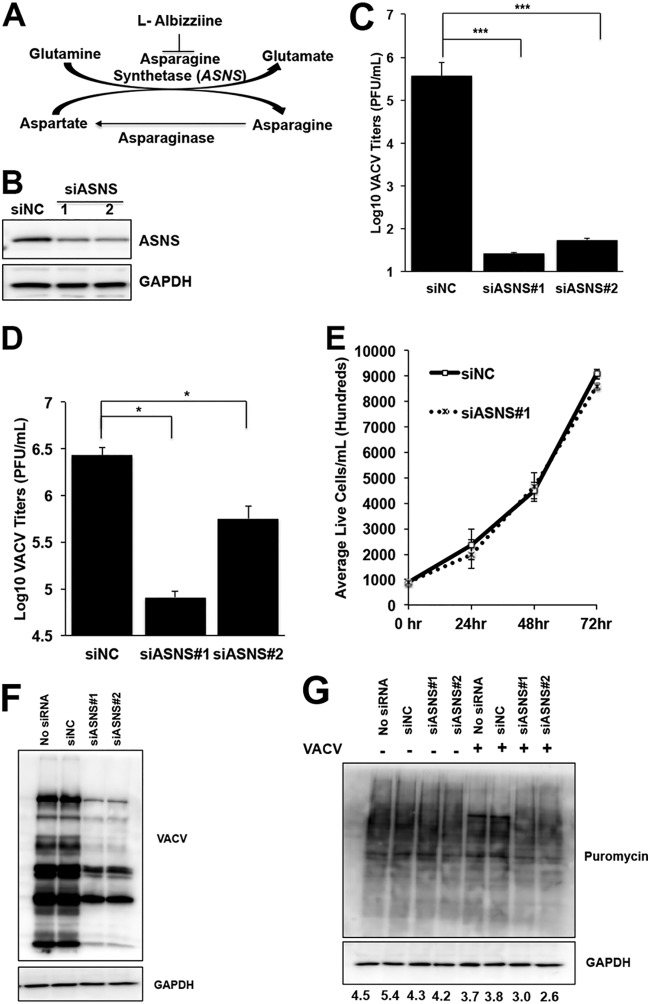
ASNS knockdown impairs VACV replication. (A) Schematic of asparagine metabolism. ASNS catalyzes the *de novo* biosynthesis of asparagine. Asparaginase catalyzes the conversion of asparagine to aspartate (inactive in mammalian cells). l-Albizziine is a competitive inhibitor of ASNS. (B) ASNS siRNAs efficiently knock down ASNS protein level. HFFs were transfected with indicated siRNA for 72 h, and the indicated proteins were detected using specific antibodies. (C, D) ASNS knockdown severely impairs VACV replication. HFFs were transfected with indicated siRNAs for 72 h and then infected with VACV at an MOI of 0.001 (C) or 2 (D). VACV titers were measured at 72 and 24 hpi, respectively, using a plaque assay. (E) ASNS knockdown does not affect the proliferation of HFFs. HFFs treated with indicated siRNAs and numbers of live cells were counted using a hemocytometer for the indicated time period. (F) ASNS knockdown impairs VACV protein synthesis. HFFs transfected with the indicated siRNAs were infected with VACV at an MOI of 2 for 24 h, and the proteins were analyzed by Western blotting using VACV antibody. (G) ASNS knockdown inhibits nascent protein synthesis in VACV-infected cells. HFFs transfected with indicated siRNAs were infected with VACV at an MOI of 2 or mock infected for 24 h. Cells were treated with 10 μg/ml puromycin for 10 min before harvesting for Western blotting using indicated antibodies. The numbers indicate GAPDH-normalized puromycin intensities. Error bars represent the standard deviation of at least three biological replicates. *, *P* ≤ 0.05; ***, *P* ≤ 0.001.

Standard cell culture medium lacks asparagine, but cells synthesize it *de novo* by ASNS, using glutamine as the amino group donor (Fig. 6A). To test whether asparagine biosynthesis affects VACV replication, ASNS protein expression was reduced with two different small interfering RNAs (siRNAs) (Fig. 6B). ASNS knockdown significantly impaired VACV replication (Fig. 6C and D), but did not suppress HFF proliferation (Fig. 6E). In ASNS siRNA-treated cells, VACV protein synthesis was downregulated (Fig. 6F). This agrees with the result that siRNA-mediated interference of ASNS also decreased nascent viral protein synthesis in VACV-infected cells (Fig. 6G). Since these experiments were performed in the presence of glutamine, the results indicate a critical role of asparagine biosynthesis in VACV replication.

### Chemically suppressing asparagine metabolism decreases VACV replication.

**FIG 7:**
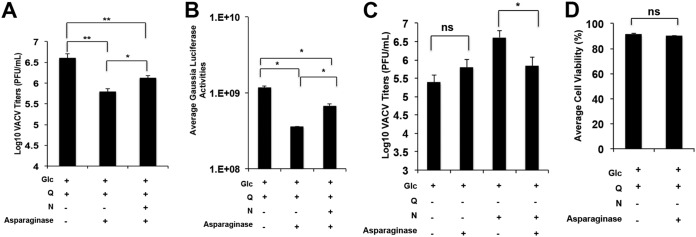
Asparaginase treatment impairs VACV replication. (A) Asparaginase treatment reduces VACV replication. HFFs were pretreated with 10 U of asparaginase for 24 h and infected with VACV at an MOI of 2 for 24 h in indicated medium. Plaque assays measured VACV titers. (B) Asparaginase treatment reduces VACV gene expression. HFFs were pretreated with 10 U of asparaginase for 24 h and infected with vLGLuc at an MOI of 2 for 16 h. Gaussia luciferase activities were measured. (C) Asparaginase reduces VACV replication in medium containing asparagine but has no effect on medium containing glucose only without asparagine and glutamine. HFFs were treated with asparaginase and infected with VACV at an MOI of 2 for 24 h in the indicated medium. Plaque assays measured VACV titers. (D) Asparaginase treatment does not reduce HFF cell viability. HFFs were treated with 10 U of asparaginase for 48 h before the cell viability was measured by trypan blue exclusion assay. Error bars represent the standard deviation of at least three biological replicates. ns, *P* > 0.05; *, *P* ≤ 0.05; **, *P* ≤ 0.01.

Conversion of asparagine to aspartate is catalyzed by asparaginase (39). To test how depleting asparagine affects VACV replication, asparaginase from E. coli was added to culture medium containing glutamine. This led to a significant decrease in VACV replication that could be partially rescued by supplementing with asparagine (Fig. 7A). Asparaginase treatment also decreased Gaussia luciferase activity in vLGluc-infected cells, which again could be partially rescued with supplemental asparagine (Fig. 7B). Although asparaginase treatment decreased VACV titers in medium containing asparagine, it did not affect viral replication in medium containing glucose only without asparagine and glutamine (Fig. 7C). Importantly, asparaginase treatment did not decrease HFF cell viability (Fig. 7D).

**FIG 8:**
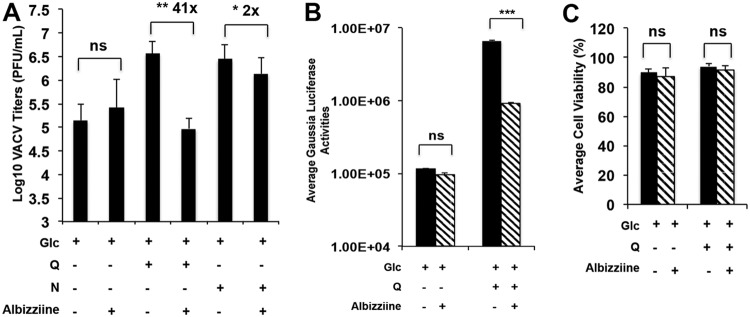
l-Albizziine treatment impairs VACV replication. (A) Inhibition of ASNS by l-albizziine reduces VACV replication in glutamine containing medium. HFFs were infected with VACV, at an MOI of 2 in the indicated medium in the presence or absence of 5 mM l-albizziine. VACV titers were measured at 24 hpi by a plaque assay. (B) Albizziine reduces Gaussia luciferase activity of recombinant VACV in glutamine-containing medium. HFFs were infected with vLGLuc at an MOI of 2 in the indicated media in the presence or absence of 5 mM l-albizziine. Gaussia luciferase activity was measured at 8 hpi. (C) Albizziine treatment does not reduce HFF cell viability. HFFs were cultured in indicated medium in the presence or absence of 10 mM l-albizziine for 24 h. Cell viability was measured by trypan blue exclusion assay. Error bars represent the standard deviation of at least three biological replicates. ns, *P* > 0.05; *, *P* ≤ 0.05; **, *P* ≤ 0.01; ***, *P* ≤ 0.001. The numbers above the bars represent fold changes.

To test whether chemical interference of ASNS impedes VACV replication, albizziine, a competitive inhibitor of ASNS (40), was added to the culture medium. Albizziine reduced VACV replication by 41-fold in medium with glutamine but had no apparent effect on VACV replication in cells grown with glucose only (Fig. 8A). In cells grown with asparagine plus glucose, albizziine reduced VACV titers by only 2-fold (Fig. 8A). Furthermore, albizziine treatment decreased Gaussia luciferase activity in vLGluc-infected HFFs grown with medium containing glutamine and glucose, but not in that containing glucose only (Fig. 8B). Albizziine did not affect HFF cell viability (Fig. 8C). Overall, these findings again demonstrate that interfering with asparagine metabolism suppresses VACV replication.

## DISCUSSION

**FIG 9:**
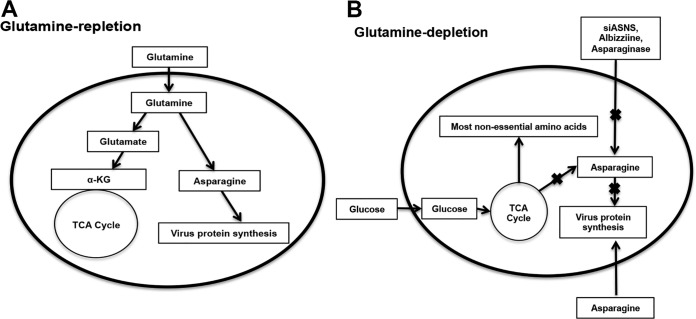
Proposed model for the role of asparagine in VACV replication. (A) In VACV-infected cells under glutamine-replete conditions, glutamine supports the TCA cycle activities (glucose is dispensable) and also allows asparagine *de novo* biosynthesis that promotes viral protein synthesis. (B) Under glutamine-depleted conditions or other ways in which the asparagine supply is affected, VACV postreplicative protein synthesis is inhibited, although glucose is able to sustain the TCA cycle activities and biosynthesis of most other nonessential amino acids.

This study used a combination of nutrient manipulation and genetic and chemical interference to establish that asparagine is a critical limiting amino acid for VACV protein synthesis that accounts for glutamine dependency of VACV replication. During VACV infection in glutamine-containing medium, glutamine not only feeds the TCA cycle but also acts as a substrate for asparagine biosynthesis to support VACV replication (Fig. 9A). Since the *de novo* synthesis of asparagine uses glutamine as the amino group donor, asparagine cannot be sufficiently synthesized in the absence of exogenous glutamine—this renders asparagine a limiting metabolite that can be compensated by exogenous supply (Fig. 9B). In the absence of glutamine, a carbon source like glucose is required to feed the TCA cycle (Fig. 9B; in mammalian cells, asparagine is unable to feed the TCA cycle by converting to aspartate). When asparagine supply is blocked by chemical or genetic interference, VACV replication is suppressed, supporting our idea that asparagine is a limiting factor of VACV replication (Fig. 9B). Although glutamine can contribute to the biosynthesis of several nonessential amino acids via glutamate, asparagine biosynthesis exclusively requires glutamine (23, 24), which is consistent with the result that rescue of VACV replication from glutamine depletion is specific to asparagine.

Glucose and glutamine are two common carbon sources for mammalian cells (41, 42). Previous studies have established that VACV prefers glutamine to glucose for efficient replication (14, 15). Yet, supplementation of α-KG or glutamate only partially rescued VACV replication from glutamine depletion (14, 15) (Fig. 1B), and glutaminase inhibition had a more severe effect on VACV replication in the absence of glucose (Fig. 2C). These results indicate that the utilization of glutamine to feed the TCA cycle only partially accounts for its role during VACV replication and is not the cause of the glutamine preference during VACV replication. Our results identified asparagine biosynthesis as a critical function that glutamine provides for efficient VACV replication. Thus, glutamine provides both the functions of glucose and asparagine required during VACV infection. Interestingly, Greseth et al. showed that *de novo* fatty acid biosynthesis is important for efficient VACV replication (14). It would be interesting to examine whether asparagine is required for efficient fatty acid synthesis during VACV replication in the future.

Asparagine mainly functions through its availability as a limiting nutrient for VACV postreplicative protein synthesis. This is supported by the evidence showing an overall accumulation of most of the amino acids under the glutamine depletion condition during VACV infection, suggesting that they can be supplied by glucose metabolism. In contrast, asparagine does not accumulate, and supplemental asparagine reduced the accumulation of amino acids under the glutamine-depleted condition. Interestingly, knockdown of ASNS or glutamine depletion has little effect on cell proliferation in uninfected HFFs, although the nascent cellular protein synthesis decreases, which is likely because the demand of nascent protein synthesis in uninfected HFFs is lower due to preexisting proteins in cells. While the limited availability of asparagine in glutamine depletion has no effect on uninfected cell proliferation, the suppression is magnified during the postreplicative stage of VACV protein synthesis. Three mechanisms can contribute to the suppression of the virus after viral DNA replication, but not during early protein synthesis, due to the shortage of asparagine supply. First, there is an increased demand of nascent protein synthesis during the late time of VACV infection to produce a large number of viral particles. Second, VACV proteins have an almost 100% higher asparagine content than human proteins. Although asparagine contents are higher in all VACV early and postreplicative (intermediate and late) proteins comparing to human proteins, the postreplicative proteins are expressed at much higher levels to build the viral particles (33, 34), which explains the suppression of viral protein synthesis is at the postreplicative stage of VACV infection. These two reasons, as discussed above, can lead to an exhaustion of asparagine supply in VACV-infected cells late during infection. Finally, the shortage of asparagine supply in glucose-only medium creates an amino acid imbalance that causes an upregulation of eIF2α phosphorylation in VACV-infected cells. We are aware that the eIF2α phosphorylation changes are not high. However, it has been suggested that small changes in eIF2α phosphorylation may cause a significant effect on overall mRNA translation rates due to the limited supply of eIF2B (37). Therefore, it is likely that the eIF2α phosphorylation upregulation can reinforce the suppression of VACV postreplicative protein synthesis in glucose-only medium. It is likely that these three mechanisms synergize to exert the outcome of viral postreplicative protein synthesis suppression. Since many viral infections demand rapid and robust nascent protein synthesis to build up viral particles, asparagine might also be a limiting metabolite in the replication of other viruses.

Emerging evidence demonstrates that asparagine plays a unique and specialized role in regulating various biological processes and disease development in mammalian cells, in addition to being a simple protein building block (31, 43–45). Asparagine biosynthesis and metabolism are evolutionarily tailored in mammalian cells so that its supply is limited and highly regulated. ASNS is the only enzyme to catalyze asparagine *de novo* synthesis. In glucose metabolism, asparagine is a nonessential amino acid to be synthesized at the very end of the TCA cycle, and the synthesis is exclusively glutamine dependent (Fig. 1A). Unlike in flies and worms, in mammalian cells asparagine is not used to feed the TCA cycle by converting to aspartate (mammalian cell asparaginase is inactive) for reasons not completely understood (46, 47). These unusual features render asparagine an attractive target in studying disease development and treatment. To this end, asparagine has received increasing attention recently, especially for its essential role in cancer development. A recent study indicates that asparagine controls breast cancer metastasis in an animal model (27). Asparagine is also important for cancer cell proliferation in multiple cancer cells, especially in the absence of glutamine, due to the requirement for various functions of asparagine (23, 24, 28, 29, 48). The crucial role of asparagine bioavailability in cancer development might, at least partly, account for its limited supply in mammalian cells. Asparagine metabolism is also critical in vessel formation (48). This, together with its importance for VACV replication, suggests that asparagine metabolism is a critical limiting factor in multiple biological processes and diseases, which highlights the importance of studying metabolic regulation of asparagine to understand its roles in various life processes.

Asparagine metabolism can serve as an attractive target for novel antipoxvirus strategy development. In fact, l-asparaginase has been used for decades to treat various cancers, including acute lymphoblastic leukemia (ALL), acute myeloid leukemia (AML), and non-Hodgkin's lymphoma (49–52). In the future, it would be interesting to investigate how modulation of asparagine metabolism affects poxvirus infection in an animal model. Furthermore, its role in cancer cell proliferation and cancer development implicates asparagine metabolism as a target for designing improved poxvirus-based cancer therapies.

## MATERIALS AND METHODS

### Cells and viruses.

Primary human foreskin fibroblasts (HFFs; kindly provided by Nicholas Wallace, Kansas State University) were cultured in Dulbecco’s minimal essential medium (DMEM; Fisher Scientific) supplemented with 10% fetal bovine serum (FBS; Peak Serum), 2 mM glutamine (VWR), 100 U/ml of penicillin, and 100 μg/ml streptomycin (VWR). BS-C-1 cells (ATCC CCL-26) were grown in Eagle’s minimal essential medium (EMEM; Fisher Scientific) supplemented with 10% FBS, 2 mM glutamine, 100 U/ml of penicillin, and 100 μg/ml streptomycin. All cells were incubated at 37°C in an incubator with 5% CO_2_. VACV Western Reserve (WR) strain (ATCC VR-1354) was used in this study. Amplification, purification, infection, and titration of VACV were carried out using methods described elsewhere (53). Recombinant VACVs encoding a Gaussia luciferase gene under an early (C11R, vEGluc), intermediate (G8R, vIGluc), or late (F17R, vLGluc) promoter were constructed by Jason Laliberte at the National Institute of Allergy and Infectious Diseases (NIAID) and generously provided by Bernard Moss (NIAID). Recombinant VACV encoding a green fluorescent protein (GFP) was described elsewhere (54).

### Antibodies and chemicals.

l-Glutamate, l-aspartate, l-serine, l-proline, and l-alanine were purchased from VWR. l-Asparagine, dimethyl 2-oxoglutarate (dimethyl α-ketoglutarate), l-methionine sulfoximine (L-MSO), Asparaginase and puromycin were purchased from Sigma-Aldrich. Dimethyl sulfoxide (DMSO) and l-albizziine were purchased from Thermo Fisher Scientific. EmbryoMax nucleoside (100×) solution was purchased from EMD Millipore. Calyculin A was purchased from Santa Cruz Biotechnology.

Anti-GCN2 (Phos T899) and anti-glyceraldehyde-3-phosphate dehydrogenase (anti-GAPDH) antibodies were purchased from Abcam. Antibodies against GCN2, phospho-eIF2α (Ser51), and total eIF2α were purchased from Cell Signaling Technology. ASNS antibody was purchased from Proteintech. Antipuromycin antibody was purchased from Sigma-Aldrich. Antibodies against the whole VACV viral particle were kindly provided by Bernard Moss.

### Glutamine depletion and rescue.

For glutamine depletion, special DMEM (Fisher Scientific) was used; it lacks glucose, l-glutamine, sodium pyruvate, and phenol red. This medium also lacks l-asparagine. The medium was supplemented with 2% dialyzed FBS (Fisher Scientific) to thoroughly deplete small molecules and amino acids while still providing other essential factors for cell growth. For glutamine depletion and rescue experiments, 1 g/liter glucose (Fisher Scientific), 2 mM glutamine, and 2 mM l-asparagine or other metabolites were added to the medium when necessary. The cells were washed with 1× phosphate-buffered saline (PBS; VWR) prior to VACV infection.

### Global metabolic profiling.

HFFs were grown in T-175 flasks. At about 95% to 100% confluence, they were washed twice with 1× PBS and then infected in different media with VACV at an MOI of 3. At 8 hours postinfection (hpi), the cells were harvested by scraping, and the pellet was washed twice with ice-cold PBS. The pellet was then dissolved in the extraction solvent (methanol) and stored at −80°C until shipment to Metabolon, Inc. (Durham, North Carolina), for metabolic profiling. All of the metabolic profiling experiments were performed with four biological replicates.

Proprietary analytical procedures were carried out to ensure high-quality data after minimizing the system artifacts, misassignments, and background noise among the samples. The raw reads were first normalized in terms of raw area counts, and then each biochemical was rescaled to set the median equal to one. Then, missing values were imputed with the minimum. Values for each sample were normalized by Bradford protein concentration in each sample. Each biochemical was then rescaled to set the median equal to one, and again missing values were imputed with the minimum. Three-way analysis of variation (ANOVA) with contrast tests was performed to calculate the fold change of metabolites.

### Cell viability assays.

For the trypan blue exclusion assay, cell viability was measured as described elsewhere (55). Briefly, after treatment, cells of each well (12-well plate) were treated with 300 μl of trypsin and resuspended with 500 μl of DMEM by pipetting. A 20-μl aliquot of cell suspension was gently mixed with 20 μl of 4% trypan blue (VWR). The numbers of living and dead cells were counted using a hemocytometer. A 3-(4,5-dimethylthiazol-2-yl)-2,5-diphenyltetrazolium bromide (MTT) cell proliferation assay (Cayman Chemicals) was performed according to the manufacturer’s instructions. Briefly, equal numbers of cells were seeded in a 96-well plate and allowed to grow overnight in a 37°C incubator, followed by necessary treatments and absorbance measurement at 570 nm using a microplate reader.

### Gaussia luciferase assay.

Cells were infected with recombinant VACV encoding a Gaussia luciferase gene. The activities of Gaussia luciferase in culture medium were measured at indicated hpi using a Pierce Gaussia luciferase flash assay kit (Thermo Scientific) and a luminometer.

### Western blotting.

The procedure was described elsewhere with minor modifications (56). For Western blotting, cells were collected and lysed using radioimmunoprecipitation assay (RIPA) lysis buffer (150 mM NaCl, 1% NP-40, and 50 mM Tris-Cl [pH 8.0]). Cell lysates were reduced by 100 mM dithiothreitol (DTT) and denatured by sodium dodecyl sulfate-polyacrylamide gel electrophoresis (SDS-PAGE) loading buffer and boiling for 5 min before SDS-PAGE. After electrophoresis, the proteins were transferred to a polyvinylidene difluoride membrane (Fisher Scientific). The membrane was then blocked in Tris-buffered saline (TBS)-Tween (TBST; 50 mM Tris-HCl [pH 7.5], 200 mM NaCl, and 0.05% Tween 20) containing 5% bovine serum albumin (BSA; VWR) for 1 h, incubated with primary antibody in the same TBST/BSA buffer for 1 h, washed with TBST three times for 10 min/each time, incubated with horseradish peroxidase-conjugated secondary antibody for 1 h, washed three times with TBST, and developed with chemiluminescent substrate (National Diagnostics). The whole procedure was carried out at room temperature. Antibodies were stripped from the membrane by Restore buffer (Thermo Fisher Scientific) for Western blotting using another antibody.

### Nascent protein synthesis analysis.

To label the newly synthesized proteins, the puromycin labeling-based SUrface SEnsing of Translation (SUnSET) method was used. This method allows for detection of protein synthesis in whole-cell lysates using Western blotting and can be used as a newly developed valid alternative to using traditional radioactive isotopes to label nascent protein synthesis (57, 58). Briefly, 10 μg/ml of puromycin (Sigma-Aldrich) was added to the cells 10 min prior to sample collection. The cells were then harvested for immunoblotting using antipuromycin antibody.

### Real-Time PCR.

Total RNA was extracted using TRIzol reagent (Ambion), followed by purification using an Invitrogen PureLink RNA minikit (Thermo Fisher Scientific). The RNA was used to synthesize cDNA using a SuperScript III first-strand synthesis kit (Invitrogen) according to the manufacturer’s instructions and using random hexamer primers. Relative mRNA levels were quantified by the CFX96 real-time PCR instrument (Bio-Rad) with All-in-One 2× quantitative PCR (qPCR) mix (GeneCopoeia) and primers specific for indicated genes. The qPCR program was started with an initial denaturation step at 95°C for 3 min, followed by 40 cycles of denaturation at 95°C for 10 s, annealing and reading fluorescence at 52°C for 30 s, and extension at 72°C for 30 s. 18S rRNA was used as a normalization factor for different samples.

### RNA interference.

Specific and negative-control siRNAs (siNC) were purchased from Integrated DNA Technologies (IDT). HFFs were transfected at a concentration of 5 nm using lipofectamine RNAiMAX (Fisher Scientific), according to the manufacturer’s instructions. Knockdown efficiency was measured by Western blotting of protein levels.

### Amino acid content calculation.

The amino acid sequence of proteins encoded by Western Reserve VACV (GenBank accession number NC_006998) was downloaded from the NCBI database. The proteins were classified as early, intermediate, or late based on previous publications (59, 60). The amino acid sequences of 20,404 human proteins that were reviewed and manually annotated from literature and curator-evaluated computational analysis were downloaded from UniProt. The asparagine content of the proteins was calculated using the ExPASy ProtParam tool (61).

### Statistical analysis.

Unless otherwise stated, the data represented are the mean of at least three biological replicates. For the analyses of global metabolic profiling, four biological replicates were used for each treatment, and the data were analyzed in R Studio (version 1.1.442). A two-tailed paired *t* test was used to evaluate significance in the difference between two means. Error bars represent the standard deviation of the experimental replicates. The following convention for symbols is used to indicate statistical significance: ns, *P* > 0.05; *, *P* ≤ 0.05; **, *P* ≤ 0.01; ***, *P* ≤ 0.001; ****, *P* ≤ 0.0001.

## Supplementary Material

Supplemental file 1
