# Supplementary material for: Asparagine Is a Critical Limiting Metabolite for Vaccinia Virus Protein Synthesis during Glutamine Deprivation
Source: J Virol. 2019 Jun 14;93(13):e01834-18. doi: 10.1128/JVI.01834-18 (PMC6580962; doi:10.1128/JVI.01834-18)
Supplement: Supplemental file 1 [file JVI.01834-18-s0001.pdf]

Supplementary Table S1. Levels of various metabolites upon infection of HFFs with VACV. Samples were prepared as described in materials and methods. Global metabolic profiling was done by Metabolon Inc. (Durham, NC) using proprietary techniques. The mean values of the metabolites of four biological replicates detected by global metabolic profiling after normalizing the data with BCA protein assay is shown.

			Mean Values		
**Super Pathway**	**Sub Pathway**	**Biochemical Name**	**Infected 8hr Glucose**	**Infected 8hr Glucose + Asparagine**	**Infected 8hr Glucose + Glutamine**
Amino Acid	Glycine, Serine and Threonine Metabolism	glycine	1.8818	1.2362	0.8507
		N-acetylglycine	1.5295	1.0690	0.8637
		betaine	1.5974	1.1713	0.8993
		serine	2.2930	1.2953	0.8544
		N-acetylserine	2.0188	0.9825	0.5863
		threonine	2.4555	1.3971	0.7191
		N-acetylthreonine	1.9251	1.0229	0.7870
		homoserine lactone	1.8493	1.5705	0.6175
	Alanine and Aspartate Metabolism	alanine	1.0758	0.9882	2.6772
		N-acetylalanine	2.0497	1.1565	0.9972
		aspartate	1.5420	0.5916	9.1995
		N-acetylaspartate (NAA)	1.6326	0.9966	0.9081
		asparagine	0.6367	36.2614	0.8955
		N-acetylasparagine	1.4213	1.0842	0.4343
	Glutamate Metabolism	glutamate	1.9136	1.1897	7.3297
		glutamine	1.1028	1.1637	56.6077
		alpha-ketoglutaramate*	0.4252	0.7239	9.1441
		N-acetylglutamate	1.7724	1.1766	0.8636
		N-acetylglutamine	1.6627	0.9289	2.1403
		gamma-carboxyglutamate	1.6299	0.9594	0.9563
		glutamate, gamma-methyl ester	1.5325	1.2830	6.3971
		pyroglutamine*	1.6711	1.5886	2.9939
		N-acetyl-aspartyl-glutamate (NAAG)	1.3366	0.9357	0.6884
		beta-citrylglutamate	1.2068	0.8205	0.7509
		carboxyethyl-GABA	0.9279	0.4257	0.3889
		N-methyl-GABA	1.5991	0.8610	0.8796
		S-1-pyrroline-5-carboxylate	1.1309	0.8027	4.6471
	Histidine Metabolism	histidine	1.8656	0.9971	0.5691
		N-acetylhistidine	1.4502	0.4984	0.3503

		imidazole propionate	0.4556	1.6186	2.1862
		imidazole lactate	2.8831	1.2115	0.3007
		carnosine	1.6698	1.0648	0.8985
		4-imidazoleacetate	1.7973	1.2298	0.7754
	Lysine Metabolism	lysine	1.1148	0.9473	0.7268
		N6,N6,N6-trimethyllysine	2.3720	0.9445	0.5136
		5-(galactosylhydroxy)-L-lysine	0.8254	0.6146	1.0282
		saccharopine	2.3161	0.9209	0.9011
		2-aminoadipate	2.5072	0.9590	0.2661
		pipecolate	3.5884	1.7770	0.1115
		6-oxopiperidine-2-carboxylate	1.6278	0.7307	0.6811
		5-aminovalerate	3.0547	1.5832	1.1345
		N,N,N-trimethyl-5-aminovalerate	1.6073	1.0161	0.7976
	Phenylalanine Metabolism	phenylalanine	1.6667	1.0552	0.6943
		N-acetylphenylalanine	1.2333	0.7376	0.6207
		1-carboxyethylphenylalanine	1.7908	1.1093	0.5119
		phenyllactate (PLA)	1.8874	0.8388	0.3236
	Tyrosine Metabolism	tyrosine	1.7995	1.0601	0.6242
		4-hydroxyphenylpyruvate	0.9099	0.7065	0.4521
		3-(4-hydroxyphenyl)lactate	3.2315	1.1975	0.0721
		O-methyltyrosine	0.9164	0.6126	0.7328
		N-formylphenylalanine	0.9526	0.7122	0.9469
	Tryptophan Metabolism	tryptophan	1.8578	1.0098	0.4685
		C-glycosyltryptophan	1.0127	0.6727	1.0000
		kynurenine	1.1125	0.9273	5.3861
		indolelactate	3.0800	1.0263	0.0451
	Leucine, Isoleucine and Valine Metabolism	leucine	1.5522	1.0022	0.7143
		4-methyl-2-oxopentanoate	0.7605	1.0796	0.8464
		isovalerylcarnitine (C5)	1.7726	0.8765	0.6589
		beta-hydroxyisovalerate	0.6704	0.5377	0.6956
		isoleucine	1.3854	0.9191	0.7074
		N-acetylisoleucine	0.9390	0.6026	0.6093
		3-methyl-2-oxovalerate	0.6616	0.9783	0.7976
		2-methylbutyrylcarnitine (C5)	1.3643	1.0999	0.5150
		methylsuccinate	0.6389	0.7469	0.7395
		valine	1.5244	1.0235	0.6950
		1-carboxyethylvaline	2.3207	1.2689	0.7732
		3-methyl-2-oxobutyrate	0.7430	1.0382	0.8203
		isobutyrylcarnitine (C4)	1.3287	1.1507	0.6647

Methionine, Cysteine, SAM and Taurine Metabolism	methionine	2.2557	1.2380	0.4114
	N-acetylmethionine	2.6450	1.8309	1.0624
	N-formylmethionine	2.1157	1.4269	0.8719
	methionine sulfone	0.1859	0.1883	0.5679
	methionine sulfoxide	1.1441	1.0273	4.3073
	N-acetylmethionine sulfoxide	0.9249	0.8458	1.1967
	S-adenosylmethionine (SAM)	2.0409	1.7928	0.8916
	S-adenosylhomocysteine (SAH)	2.1413	1.2588	1.0603
	homocysteine	1.6955	0.9524	0.9041
	cystathionine	2.5584	1.6184	1.4234
	cysteine	1.3037	1.1321	0.8495
	N-acetylcysteine	0.9444	0.7393	1.2352
	cystine	0.1195	0.5642	0.0499
	lanthionine	1.3975	0.9397	0.9502
	hypotaurine	4.1631	1.9340	1.5121
	taurine	3.8526	1.3187	1.2370
	N-acetyltaurine	2.2228	1.0813	0.9021
	3-sulfo-L-alanine	0.3154	0.2519	1.0256
Urea cycle; Arginine and Proline Metabolism	arginine	1.1367	0.9166	0.7339
	argininosuccinate	1.2748	0.4604	6.9681
	ornithine	0.8470	0.9759	2.2514
	2-oxoarginine*	0.5359	0.5637	0.5354
	citrulline	1.1130	1.4448	0.6518
	proline	1.4366	1.0433	1.9626
	dimethylarginine (SDMA + ADMA)	1.6497	0.9374	0.7336
	trans-4-hydroxyproline	1.0180	1.0941	0.9987
	pro-hydroxy-pro	1.4301	0.9723	1.7912
Creatine Metabolism	creatine	1.4440	1.0483	0.9076
	creatinine	1.8121	1.0884	0.9503
	creatine phosphate	2.3719	1.0486	0.9731
Polyamine Metabolism	putrescine	0.4104	1.3321	1.2023
	spermidine	1.3006	1.3371	0.7414
	5-methylthioadenosine (MTA)	2.3509	1.6771	0.9509
	N-acetylputrescine	0.7944	2.5626	2.0039
	(N(1) + N(8))-acetylspermidine	1.8852	1.9526	1.1059
Guanidino and Acetamido Metabolism	4-guanidinobutanoate	1.1934	0.7431	3.5653
Glutathione Metabolism	glutathione, reduced (GSH)	4.1123	2.0947	4.7345

		glutathione, oxidized (GSSG)	1.6414	1.0133	1.7158
		cysteine-glutathione disulfide	0.4830	0.8494	0.1935
		S-methylglutathione	5.9649	1.8556	1.9624
		S-lactoylglutathione	2.1245	3.3003	4.6601
		cysteinylglycine	3.6614	1.9740	3.1225
		5-oxoproline	0.5617	0.9949	5.3714
Peptide	Gamma-glutamyl Amino Acid	gamma-glutamylalanine	1.5640	1.0507	1.1563
		gamma-glutamylcysteine	1.0114	0.4806	4.2390
		gamma-glutamylglutamate	2.7709	1.1745	5.5500
		gamma-glutamylglutamine	0.0411	0.0411	1.1389
		gamma-glutamylisoleucine*	2.1627	0.9513	1.0152
		gamma-glutamyl-epsilon-lysine	0.6843	0.5420	0.9686
		gamma-glutamylthreonine	1.6045	1.1047	1.7267
		gamma-glutamylvaline	1.4313	0.8510	1.3570
		gamma-glutamyl-2-aminobutyrate	1.6053	0.7296	0.4786
	Dipeptide	leucylglycine	0.5033	0.8135	1.0203
		phenylalanylglycine	0.6284	0.6430	0.8585
	Acetylated Peptides	phenylacetylglycine	1.3107	1.1526	0.5943
Carbohydrate	Glycolysis, Gluconeogenesis, and Pyruvate Metabolism	glucose	0.7137	1.1001	0.9387
		glucose 6-phosphate	0.8806	0.5645	0.6546
		fructose 1,6-diphosphate/glucose 1,6-diphosphate/myo-inositol diphosphates	0.7828	0.8326	1.2205
		dihydroxyacetone phosphate (DHAP)	0.8555	0.6433	0.9006
		2-phosphoglycerate	1.3084	0.3762	0.6612
		3-phosphoglycerate	0.3825	0.4804	0.9529
		phosphoenolpyruvate (PEP)	0.3916	0.3904	1.0609
		pyruvate	0.6244	0.6954	1.3269
		lactate	0.5178	0.8311	0.6825
		glycerate	0.7853	0.6166	1.1012
	Pentose Phosphate Pathway	6-phosphogluconate	0.6747	0.8684	1.0248
		sedoheptulose-7-phosphate	1.4886	0.4947	0.2602
	Pentose Metabolism	ribitol	2.7864	1.3731	0.6256
		ribonate	2.7754	0.9796	0.6638
		arabitol/xylitol	2.2754	1.1125	0.6655
		arabonate/xylonate	1.4631	0.6750	0.4987
		ribulonate/xylulonate*	0.6831	1.2017	1.0189
	Glycogen Metabolism	maltotetraose	0.2941	0.2941	0.7799
	Disaccharides and Oligosaccharides	sucrose	1.1254	1.1372	0.9855

	Fructose, Mannose and Galactose Metabolism	fructose	0.6304	1.5168	3.0039
		mannitol/sorbitol	1.5420	1.6320	0.9433
		mannose	0.7000	0.8728	1.3429
		galactonate	2.7641	1.2744	0.7581
	Nucleotide Sugar	UDP-glucose	1.4234	0.8289	0.6762
		UDP-galactose	1.4279	0.7916	0.7191
		UDP-glucuronate	1.4430	0.4788	0.2795
		guanosine 5'-diphospho-fucose	1.1094	0.5542	0.6528
		UDP-N-acetylglucosamine/galactosamine	1.3927	0.8679	0.8061
		cytidine 5'-monophospho-N-acetylneuraminic acid	1.5704	0.6240	0.9416
	Aminosugar Metabolism	glucuronate	1.5262	1.1837	1.1946
		N-acetylneuraminate	0.9946	0.6826	0.9556
		N-acetylglucosaminylasparagine	2.0310	1.0993	0.8912
		erythronate*	1.8187	1.0250	0.8481
		N-acetylglucosamine/N-acetylgalactosamine	0.5775	0.6205	1.0179
Energy	TCA Cycle	citrate	1.7202	1.0020	1.1582
		aconitate [cis or trans]	1.6671	0.8184	1.1169
		alpha-ketoglutarate	1.7781	0.9625	4.6373
		succinate	0.7641	0.7835	1.7204
		fumarate	1.2650	1.0564	12.9296
		malate	1.3126	0.9793	15.7390
		2-methylcitrate/homocitrate	0.6596	0.5954	0.4071
	Oxidative Phosphorylation	phosphate	0.8344	0.8118	0.7642
